# Uncommon Presentation of Primary Bladder Signet Ring Cell Carcinoma With Peritoneal Carcinomatosis: A Rare Case Report

**DOI:** 10.1155/criu/5584765

**Published:** 2026-02-03

**Authors:** Jean Paule Joumaa, Kelly Katherine Karam, Joya Ghaleb, Hilda E. Ghadieh, Alaa Korhani

**Affiliations:** ^1^ Department of Biomedical Sciences, Faculty of Medicine and Medical Sciences, University of Balamand, Tripoli, Lebanon, balamand.edu.lb; ^2^ Department of Urology, Hospital Nini, Tripoli, Lebanon

**Keywords:** bladder cancer, immunohistochemistry, PD-L1, peritoneal carcinomatosis, signet ring cell carcinoma, urinary incontinence

## Abstract

Primary signet ring cell carcinoma (SRCC) of the bladder is an exceptionally rare and aggressive malignancy, accounting for only 0.12%–0.6% of all bladder cancers. This case report describes a 54‐year‐old female who presented with urinary incontinence and abdominal pain, initially misdiagnosed as a urinary tract infection. Imaging revealed suspicious bladder findings, and subsequent cystoscopy with transurethral resection identified SRCC, later confirmed by immunohistochemistry (PD‐L1 positive, CDX‐2/ER negative). Despite peritoneal carcinomatosis, the patient responded to cisplatin/gemcitabine chemotherapy and immunotherapy, demonstrating tumor shrinkage on follow‐up imaging. This case highlights the diagnostic challenges of SRCC due to its nonspecific symptoms and potential histological overlap with other metastatic gastrointestinal tumors. Early recognition and a multidisciplinary approach are critical for improving patient outcomes.

## 1. Introduction

Primary signet ring cell carcinoma (SRCC) of the bladder is a rare and aggressive malignancy, often diagnosed at advanced stages due to its nonspecific urinary symptoms and histological resemblance to other metastatic gastrointestinal tumors (1). SRCC accounts for < 1% of bladder cancers and typically affects patients over 40 years old, with a male predominance (2). Patients commonly present with hematuria, dysuria, or urinary retention, mimicking benign conditions like urinary tract infections (3).

Histologically, SRCC is characterized by cytoplasmic mucin accumulation displacing nuclei peripherally, forming the classic “signet ring” appearance as its name states (4). Diagnosis requires exclusion of metastatic origins, particularly gastric or colorectal primary cancers, through both endoscopic and immunohistochemical evaluation (5). Management remains challenging, with radical cystectomy (RC) as the gold standard for localized disease, though advanced cases often require chemotherapy and immunotherapy (6). This case highlights the diagnostic complexities and emphasizes the need for early suspicion in patients with persistent urinary symptoms.

## 2. Case Report

A 54‐year‐old Gravida 6 Para 6 (G6P6) postmenopausal female, previously healthy, presented for urinary incontinence and lower abdominal pain. History goes back to 4 months, when the patient started having symptoms of urinary dribbling, increased frequency of urination, and dysuria, alongside a persistent lower abdominal pain. Notably, the patient has a smoking history of 30 pack‐years (one pack per day for 30 years). To rule out an infectious cause, multiple urinalyses were ordered, each showing a range of 20–25 white blood cells (WBCs) in the urine sample. A urine culture revealed *Streptococcus* species. As such, she was given the antibiotic amoxicillin and clavulanic acid, which only partially helped relieve her symptoms. As a result, a pelvic ultrasound was done that showed a right 7‐cm ovarian cyst. For confirmation, the patient visited a different physician who performed another pelvic ultrasound that showed no signs of the previously found ovarian cyst; nonetheless, it showed nonspecific but suspicious bladder findings. This prompted a visit to the urologist, who ordered a computed tomography (CT) scan, which the patient refused to do, and a cystoscopy.

During cystoscopy, due to suspicious and irregularly shaped mucosa, a transurethral resection of bladder tumor (TURBT) was done, and pathology results following surgery revealed SRCC of the bladder. A follow‐up positron emission tomography (PET) scan showed peritoneal carcinomatosis but no evidence of distant metastasis. As a result, a laparotomy was made to debulk the tumor. A second biopsy postlaparotomy also confirmed the results of the previous biopsy.

The patient underwent standard surgical cytoreduction of peritoneal carcinomatosis starting with a midline laparotomy. A systematic exploration of the peritoneal cavity was performed, and intra‐abdominal assessment demonstrated a diffuse distribution without any major vascular structure or deep parenchymal organ invasion, allowing for a complete resection. Debulking proceeded starting with omentectomy and resection of visible peritoneal implants along the anterior abdominal wall. Peritoneal stripping was performed, followed by excision of nodular deposits over the mesenteric surfaces and pelvic peritoneum. The remainder of the procedure followed standard technique, and the patient remained in stable condition.

Tumor cells were negative for CDX‐2 and ER but positive for PDL‐1 with a total proportion score (TPS) of 100% and a combined positive score (CPS) of 100%. Based on the presence of peritoneal carcinomatosis in the absence of nodal disease or distant visceral metastasis, the tumor was staged as T4b N0 M1b, corresponding to Stage IV bladder cancer according to the American Joint Committee on Cancer (AJCC) eighth edition. The patient had firm directives against any further urological surgical procedures, including partial cystectomy (PC) or RC, and elected to proceed with drug therapy as a mainstay treatment option.

**Figure 1:**
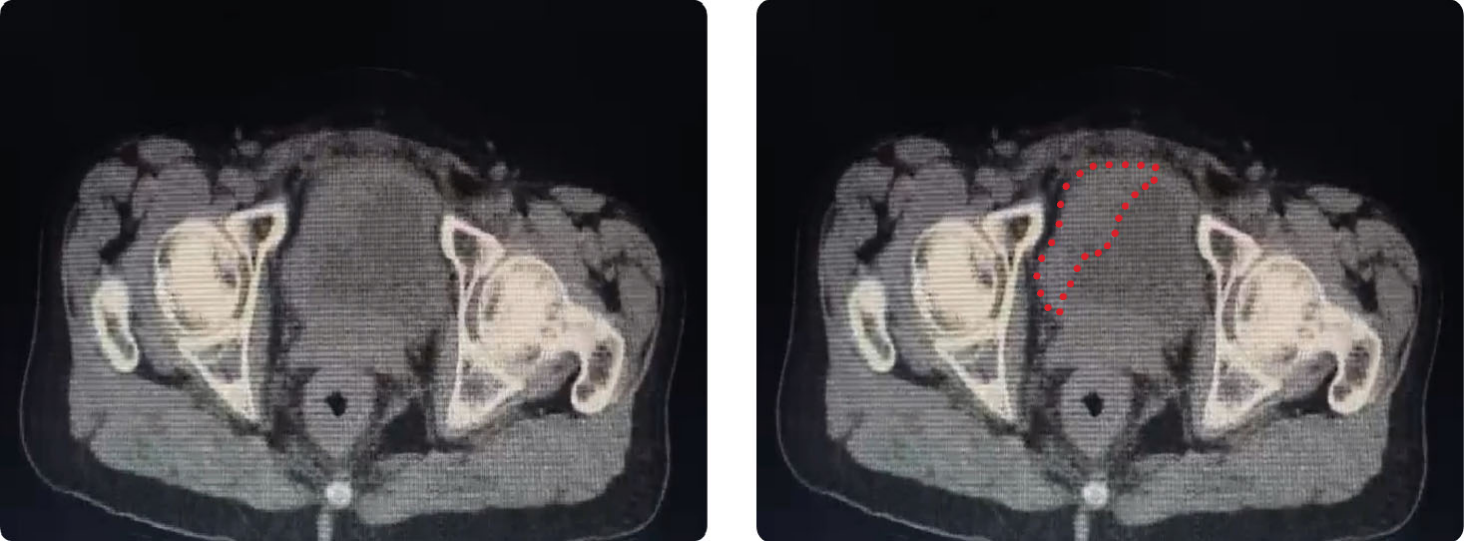
CT scan of the pelvis showing the bladder with the signet ring cell carcinoma mass (outlined in red dots) postoperatively upon 20 doses of immunotherapeutic treatment.

After a visit to the oncologist, six‐cycle chemotherapy with cisplatin and Gemzar (gemcitabine) was performed, complicated by symptoms including alopecia and a 12‐kg weight loss. Following chemotherapy, the patient was started on an immunotherapy agent, avelumab, also known as Bavencio, of which she has received 20 out of the expected 36 doses over a period of 18 months. Follow‐up abdominal and pelvic CT imaging demonstrated a reduction in tumor burden following immunotherapy (Figure 1). Cyclic scans are done every 3 months to assess tumor development. The last scan showed improvement and tumor shrinkage compared to previous scans.

## 3. Discussion

Primary SRCC of the bladder comprises 0.12%–0.6% of bladder malignancies (1). SRCC of the bladder is usually diagnosed at an advanced stage due to its rarity and aggressiveness (7). It can be found in patients of any age (2). However, its incidence increases in patients more than 40 years old, with a male‐to‐female ratio of 2.70–3.20:1 (2). SRCCs are typically found in the stomach, colon, gallbladder, or breast (8). Consequently, primary adenocarcinomas of these organs must be ruled out before the diagnosis of primary SRCC of the bladder (8).

SRCC of the bladder is a rare diagnosis and often presents at an advanced stage, making identification of its risk factors challenging. However, existing literature suggests that advanced age (> 40 years), male sex, and chronic bladder irritation or inflammation, such as cases of recurrent infections or long‐term catheter use, may predispose individuals to bladder adenocarcinomas, including SRCC subtypes (7). Genetic factors are not well defined for primary bladder SRCC, but mucin‐producing adenocarcinomas have been linked to intestinal metaplasia and chronic inflammation, which may lead to malignant transformation (9). Notably, this patient had several risk factors associated with bladder carcinoma, including a significant smoking history of 30 pack‐years, chronic urinary tract infections leading to persistent bladder inflammation, and female sex with multiparity, which may predispose to recurrent urinary dysfunction.

Bladder SRCC presents with hematuria, dysuria, frequent urination, incontinence, or urinary retention (10). These urinary symptoms are similar in all bladder cancers (3). On imaging, SRCC appears as a diffuse wall thickening in the bladder (4). However, on cystoscopy, it may appear as an exophytic growth similar to two‐thirds of other bladder carcinomas (4). In this case, the 54‐year‐old female patient′s high WBC count in urine suggested a possible urinary tract infection. The patient′s persistent urinary incontinence and lower abdominal pain prompted further investigation.

Histologically, these cancers have the appearance of a signet ring due to the accumulation of abundant mucin in the cytoplasm, leading to nuclei dislocation to the periphery (11). In this case, SRCC of the bladder was confirmed via biopsy and immunohistochemical staining. However, metastatic tumors from the gastrointestinal tract (GIT) should be eliminated before confirming the diagnosis of primary SRCC of the bladder (4). The immunostainings overlap between the GIT and bladder primary SRCC (4). Both are positive for CK7, CK20, CEA, EMA, and CDX2 (4). Nonetheless, ruling out endoscopically any GIT lesion and the absence of nuclear positivity for *β*‐catenin favors primary SRCC of the bladder (5).

The management of primary SRCC of the bladder is challenging. Surgeries such as TURBT, PC, and RC are the first treatment approach (2, 12). RC is the method of choice; however, TURBT and PC are beneficial in localized tumors (2). Although SRCC is found to be resistant to the standard chemotherapy regimens and radiotherapy used for advanced bladder carcinoma, the patient was followed by adjuvant chemotherapy with cisplatin and gemcitabine (6).

Although systematic reviews and large comparative analyses are lacking, most case reports described primary bladder SRCC as a biologically aggressive and highly lethal tumor. For instance, Jin et al. contrasted SRCC with more common urothelial carcinomas (UCs), stating that the prognosis of SRCC is poorer than that of UC, even after adjustment for patient baseline demographic and clinicopathological characteristics, as well as cancer treatment (13). SRCC often presents in advanced stages and exhibits resistance to conventional therapy.

This case emphasizes the importance of early recognition of primary SRCC of the bladder, especially in females. Bladder primary SRCC′s delayed diagnosis, high mortality, and poor prognosis underscore the need for a multidisciplinary team, including urologists, oncologists, and pathologists, to diagnose and efficiently treat it. Table 1 summarizes selected published cases, highlighting variations in patient demographics, presenting symptoms, disease stage, treatment approaches, and outcomes.

**Table 1:** Summary of published cases of primary signet ring cell carcinoma of the urinary tract.

**Study/year**	**Patient (age, sex)**	**Presentation/symptoms**	**Stage at presentation**	**Treatment**	**Prognosis**
Zeng and Swee, 2022	64, M	Bone metastases; severe hypocalcemia	Metastatic SRCC with bone mets	Chemotherapy + immunotherapy; denosumab; palliative care	Died 16 months postdiagnosis
Yamamoto et al., 2001	56, M	Oliguria; postrenal failure due to bladder tumor	pT3b, pN2, pMx	Total cystectomy; declined adjuvant chemotherapy	Died 8 months postoperatively
Celik et al., 2014	66, M	Urinary difficulty; prostate mass; extensive bone mets	Metastatic SRCC of the prostate	Hormonal therapy → docetaxel chemotherapy; palliative radiotherapy	Died of urosepsis 22 months postdiagnosis
Bouhajja et al., 2019	70, F	Right back pain, dysuria, gross hematuria	Not specified	Biopsy diagnosis; treatment not specified	Alive 3 years postdiagnosis
Bouhajja et al., 2019	53, M	Gross hematuria; bladder wall thickening	Muscle‐invasive; perivesical fat invasion	TURBT only; no cystectomy	Alive 2 years postdiagnosis

## 4. Conclusion

This case illustrates the diagnostic and therapeutic challenges of primary SRCC of the bladder, a malignancy often misdiagnosed as other benign urinary conditions. The patient′s initial misdiagnosis underscores the importance of considering SRCC in refractory urinary symptoms, particularly in older adults. Immunohistochemistry, such as PD‐L1 positivity, and imaging play important roles in confirming diagnosis and guiding therapy.

While SRCC carries a poor prognosis due to its aggressive nature and resistance to many conventional therapies, this case demonstrates that multidisciplinary management, such as combining surgery, chemotherapy, and immunotherapy, can achieve meaningful responses. Future efforts should focus on standardized diagnostic criteria and novel targeted therapies to improve survival in this highly fatal disease.

**Nomenclature**
SRCC: primary signet ring cell carcinoma
IHC: immunohistochemistry
PD‐L1: programmed death‐ligand 1
CDX‐2: caudal type homeobox 2
ER: estrogen receptor
TURBT: transurethral resection of bladder tumor
PET: positron emission tomography
TPS: total proportion score
CPS: combined positive score
Gemzar: gemcitabine
GIT: gastrointestinal tract
CK7: cytokeratin 7
CK20: cytokeratin 20
CEA: carcinoembryonic antigen
EMA: epithelial membrane antigen
PC: partial cystectomy
RC: radical cystectomy
WBCs: white blood cells

## Ethics Statement

This case report was reviewed and approved by the Institutional Review Board (IRB) of the University of Balamand, and written informed consent was obtained from the patient for the publication of this case report. All procedures followed were in accordance with the ethical standards of the responsible committee and with the Declaration of Helsinki.

## Disclosure

All authors have read and agreed to the published version of the manuscript.

## Conflicts of Interest

The authors declare no conflicts of interest.

## Author Contributions

Conception, organization, and writing of the first draft: J.P.J., K.K.K., and J.G. Writing of the first draft and editing: J.P.J., K.K.K., and J.G. Conception and design and review/editing of the manuscript: A.K. Review/editing: H.E.G., J.P.J., K.K.K., and J.G. contributed equally to these studies.

## Funding

This work did not receive specific funding.

## Data Availability

The data that support the findings of this study are available from the corresponding authors upon reasonable request.
